# Amoebae: beyond pathogens- exploring their benefits and future potential

**DOI:** 10.3389/fcimb.2024.1518925

**Published:** 2024-12-18

**Authors:** Suman Kalyan Dinda, Shreyasee Hazra, Anwesha De, Annurima Datta, Lipika Das, Santanu Pattanayak, Kishor Kumar, Manash Deep Dey, Arnab Basu, Dipak Manna

**Affiliations:** ^1^ Department of Biomedical Science and Technology, School of Biological Sciences, Ramakrishna Mission Vivekananda Educational and Research Institute (RKMVERI), Kolkata, India; ^2^ Department of Agricultural Biotechnology, Ramakrishna Mission Vivekananda Educational and Research Institute (RKMVERI), Kolkata, India; ^3^ Department of Microbiology, University of Calcutta, Kolkata, India; ^4^ Chaudhary Charan Singh Haryana Agricultural University (CCS HAU), Hisar, Haryana, India

**Keywords:** amoeba, protozoa, FLA (free-living amoeba), symbiotic, predators, primary amoebic meningoencephalitis (PAM), chronic obstructive pulmonary disease (COPD)

## Abstract

Amoebae, fascinatingly diverse protists, showcase a dual nature that positions them as both friends and foes in our world. These organisms, defined by their distinctive pseudopodia, span a spectrum from harmful to helpful. On the darker side, species like *Entamoeba histolytica* pose serious health risks, causing intestinal and liver diseases, while the infamous “brain-eating” *Naegleria fowleri* leads to fatal primary amoebic meningoencephalitis (PAM), with a daunting 97% mortality rate. Other free-living amoebae, including *Acanthamoeba castellanii* and *Balamuthia mandrillaris*, also threaten the human central nervous system. Yet, beyond these dangers, amoebae play critical ecological roles. They function as nature’s recyclers, decomposing organic material and nourishing aquatic ecosystems, while also serving as food for various organisms. Moreover, certain amoebae help control plant pathogens and offer insight into human disease, proving valuable as model organisms in biomedical research. This review sheds light on the complex, multifaceted world of amoebae, highlighting their dual role as pathogens and as key contributors to vital ecological processes, as well as their significant impact on research and their promising potential for enhancing human well-being.

## 1 Introduction

Amoebae, within the Protista kingdom, encompasses over 17,000 species, exhibiting diverse lifestyles from free-living to parasitic, thriving in both aquatic and terrestrial ecosystems, and predominantly preying on bacteria and algae, or as parasitic to humans and other animals (Rodríguez-Zaragoza, 1994; Dunn et al., 2017; Gao et al., 2019). These enigmatic creatures play multifaceted roles in nature and do not constitute a unified group; rather, they are dispersed across various eukaryotic supergroups. Many amoeboid organisms also have multiple morphologies in their lifecycles, such as flagellated forms of *N. fowleri*, reticulated forms of *Corallomyxa*, and multicellular slugs such as *Dictyostelium discoidium*, all of which play diverse roles ranging from their positive ecological contributions to their potential as threats to ecosystems and human health (Grell, 1988; Schaap, 2007; Dunn et al., 2017; Brunet et al., 2021; Kin and Schaap, 2021; Borkens, 2024). The beneficial aspects of amoebae reveal their crucial ecological importance as decomposers, thriving in aquatic environments where they break down organic matter and recycle essential nutrients (Sagova-Mareckova et al., 2021; Shi et al., 2021). Amoebae can directly and indirectly impact plants and having ability to kill plant pathogens (Long et al., 2018). Moreover, they serve as a fundamental food source for various organisms, including small fish and insects, and sustain intricate food chains (Gryseels et al., 2012; Long et al., 2018). In addition, some amoebae have developed symbiotic relationships with certain organisms, resulting in mutual benefits through nutrient exchange and protection (Shu et al., 2018; Sørensen et al., 2019). In recent years, there has been a growing acceptance of amoeba as a valuable model for investigating a wide range of human diseases (Nguyen et al., 2014; Ahmed Khan et al., 2015; Wu et al., 2020; Ghosh et al., 2022; Viennet et al., 2023). This recognition indicates the increasing relevance and utility of amoebae in biomedical research.

However, the detrimental aspects of amoebae highlight their potential to become pathogens, leading to serious health risks for both humans and animals. Notably, *E. histolytica* infections are highly prevalent in the tropical regions of the developing world, affecting millions of people. The disease can manifest as severe intestinal and liver ailments, with approximately 10% of patients experiencing life-threatening complications (Rao et al., 2009; Kantor et al., 2018).

**Figure 1:**
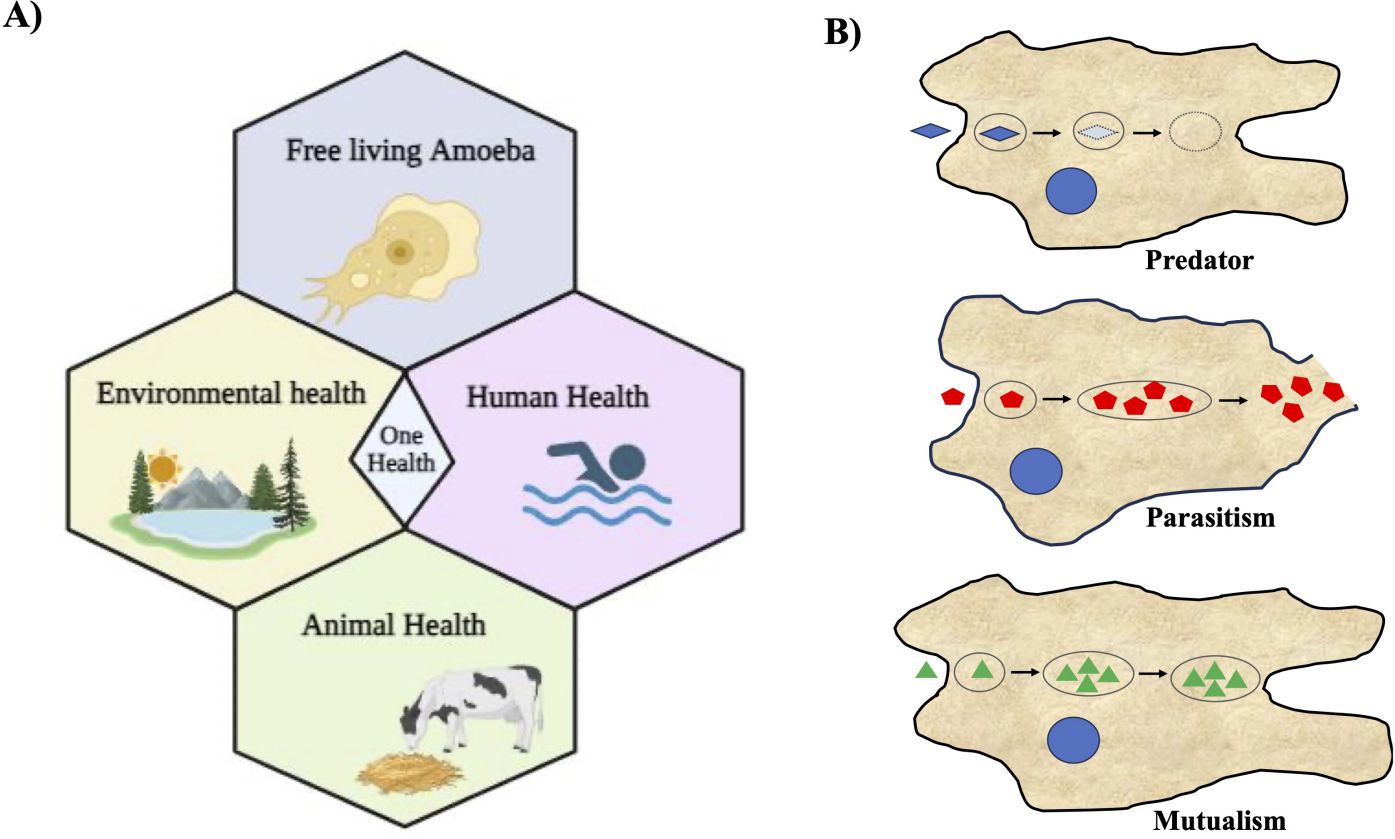
Free-living amoebae are vital in the One Health Approach and their diverse associations with microorganisms. **(A)** This illustration depicts the significance of free-living amoebae and their multifaceted implications within the context of the One Health Approach. It underscores the importance of recognizing the interplay between human, animal, and environmental health when considering the impact of these amoebae. It emphasizes the role of free-living amoebae in ecological processes and microbial communities, thereby contributing to environmental health. **(B)** Schematic illustration depicting various types of association of amoebae with other microorganisms. Three distinct types of amoeba-bacterium interactions are delineated: Predation, Parasitism, and Mutualism. In this representation, blue microorganisms symbolize prey, while red bacteria denote pathogenic species capable of replication and escape into the cytosol and the environment; and green represent the endosymbionts living within amoebae.

The unsightly aspect of amoebae becomes starkly apparent in the form of a staggering mortality rate exceeding 97%, all attributable to *N. fowleri*, also known as the ‘brain-eating amoeba,’ causes primary amoebic meningoencephalitis (PAM), a fatal human disease (Salazar-Ardiles et al., 2022). In some instances, certain amoebae can transform into opportunistic invaders, disrupting native species and biodiversity within various ecosystems (Guimaraes et al., 2016; Betanzos et al., 2019). Among the deadliest pathogens are free-living amoebae (FLA) such as *N. fowleri*, *A. castellanii*, and *B. mandrillaris* (Güémez and García, 2021). *Acanthamoeba* and *Balamuthia*, both opportunistic free-living amoebae, cause granulomatous amoebic encephalitis (GAE) and can also cause disseminated disease in immunosuppressed patients (Schafer et al., 2015; Gray et al., 2016; Kalra et al., 2020; Kofman and Guarner, 2022). *Acanthamoeba* also causes corneal infection known as *Acanthamoeba* keratitis. It has been reported that more than 80% of patients with *Acanthamoeba* keratitis use contact lenses, and the infections start from contamination of contact lenses with *Acanthamoeba* (Kalra et al., 2020; Raghavan and Rammohan, 2024). Despite being serious human pathogens, these free-living amoebae are only poorly studied from both the identification and infection perspective (epidemiological) as well as diagnostic and therapeutic (clinical) perspectives. In our ecosystem, everything including humans, animals, plants, and the environment is intrinsically interconnected, and when some intervention occurs, mainly through the action of man himself, everyone suffers the consequences. In this regard, “One Health” approaches are becoming increasingly essential in the world we live in (Leal dos Santos et al., 2022; Viani et al., 2024). Infection by *N. fowleri* is highly lethal to humans and can also infect cattle; therefore, its implications for health approaches through the One Health concept are gaining attention (**Figure 1A**). A recent report highlighted that *N. fowleri* can also infect cattle, potentially increasing infections in humans, which is a significant concern. Daft et al. reported a case of fatal meningoencephalitis caused by *N. fowleri* in Holstein cattle (Daft et al., 2005). Since *N. fowleri* can infect cattle, these animals can serve as hosts where the amoeba may multiply, potentially increasing environmental contamination and thereby the risk of human infections. This underscores the need to consider the ecological importance of interactions between amoebae and other microorganisms within the ‘One Health’ Approach, emphasizing a comprehensive health strategy (Leal dos Santos et al., 2022). Infection by *N. fowleri* is extremely lethal to humans and can also infect cattle. Therefore, implications for health approaches through the One Health concept are emerging (**Figure 1A**). Recognizing their zoonotic nature, we must consider their implications for both animal and human health when addressing these pathogens. This review explores the multifaceted nature of amoebae, focusing on their roles as both beneficial organisms and potential threats to various ecosystems and human health.

## 2 The harmful aspect of amoebae

Human *Entamoeba* infection is a parasitic disease caused by various species of the genus *Entamoeba*, with *Entamoeba histolytica* being the most common pathogenic species (Kantor et al., 2018). This infection primarily affects the gastrointestinal system and can lead to a range of clinical manifestations, including diarrhea and liver abscesses in severe cases (Johnson et al., 1968; Kagan, 1975; Matsubayashi et al., 2014; Cooper et al., 2015). *Entamoeba* is a global health concern, particularly in areas with poor sanitation and limited access to clean water (Nasrallah et al., 2022; Fu et al., 2023). It is estimated that over 50 million people worldwide are infected with *E. histolytica* (Bercu et al., 2007; Walters et al., 2019). The infection is most prevalent in tropical and subtropical regions, including parts of Africa, Asia, and Latin America. Risk factors for *Entamoeba* infection include poor sanitation and contamination of drinking water sources with human feces can facilitate the transmission of the parasite (Holtan, 1988; Mackey-Lawrence and Petri, 2011). Living conditions where living environments can increase the likelihood of person-to-person transmission of the parasite. Malnourished individuals may be more susceptible to severe forms of the disease and travelers to regions with a high prevalence of *Entamoeba* infection are at increased risk, especially if they do not follow hygiene recommendations (Petri et al., 2009; Morgado et al., 2016; Chidebelu and Nweze, 2020). *E. histolytica* possesses an array of virulence factors that enable it to colonize and invade the human host (Tillack et al., 2007; Padilla-Vaca and Anaya-Velázquez, 2010; Marie and Petri, 2014). These factors include specialized surface lectins and adhesins that facilitate adherence to and invasion of the host’s intestinal mucosa as well as the formation of durable cysts for transmission. The ability of the parasite to actively invade host tissues is aided by cysteine proteases and other enzymes that degrade host proteins, whereas its capacity to evade the host immune response is achieved through various mechanisms, such as altering cytokine profiles and phagocytosing immune cells. Furthermore, Gal/GalNAc lectin plays a pivotal role in adhesion, tissue-destructive lesions, and pathogenesis (Padilla-Vaca et al., 1999; Pacheco et al., 2004). The interplay of these virulence factors with host factors ultimately determines the outcome of *E. histolytica* infection, ranging from asymptomatic colonization to severe, potentially life-threatening diseases. Other symptoms include abdominal pain, cramping, and weight loss. Some infected individuals may remain asymptomatic carriers, shedding the parasite in their stool without experiencing any noticeable symptoms (Gathiram and Jackson, 1987; Tachibana et al., 2000; Blessmann et al., 2003).

*E. histolytica* alternates between cyst (environmentally resilient, transmittable) and trophozoite (disease-causing) stages and several transcription factors are crucial for this stage conversion (Ehrenkaufer et al., 2009; Manna et al., 2018; Manna and Singh, 2019). One of the most severe complications of *E. histolytica* infection is the development of liver abscesses (Usuda et al., 2022; Akhondi and Sabih, 2023; Jackson-Akers et al., 2023). This occurs when the parasite invades the intestinal wall and travels through the bloodstream to other organs including the liver and lungs. Abscesses can form in the liver, leading to symptoms such as fever, right upper abdominal pain, and jaundice. *Entamoeba* require high concentrations of iron for survival and reproduction. Ferritin is an iron storage protein that is mainly found in the liver and spleen of mammals. Thereby, the liver provides a plentiful source of iron, helping amoebae to multiply in that organ, making it a primary target for infection. Mortality associated with *Entamoeba* infection is primarily linked to severe cases and complications, particularly liver abscesses. If left untreated, liver abscesses can be life-threatening. Timely diagnosis and appropriate medical intervention are crucial for reducing the mortality rates associated with *E. histolytica* infection. As the world continues to face emerging infectious diseases, understanding the pathogenesis of amoeba-related diseases could help mitigate their impact. There is particular promise in the use of advanced molecular techniques, such as CRISPR-Cas9, to understand the genetic basis of pathogenicity and resistance in amoebae (Kangussu-Marcolino et al., 2021).

## 3 The unsightly aspect of amoebae

The world of amoebae is as intriguing as it is complex, showing a plethora of adaptations that have allowed them to thrive in a diverse array of environments. While their resilience and versatility often earn them admiration, there exists a less explored and rather unsettling facet – the “ugly” side of amoebae, where their adaptability can take on a sinister twist. Amoebae, seemingly innocuous single-celled organisms, have demonstrated the ability to endure and even flourish in adverse environmental conditions (Salazar-Ardiles et al., 2022). Their ability to tolerate extreme temperatures, varying levels of acidity, and even polluted or toxic environments is a testament to their tenacity (Lam et al., 2019). These adaptability to conditions that might be deemed inhospitable by many organisms underscores the remarkable nature of the amoebae. It is almost as if they hold the blueprint for survival, adapting their morphology and physiological processes to navigate through challenges that would prove fatal to others (Salazar-Ardiles et al., 2022). Certain amoebae, under specific circumstances, can exploit their adaptive processes to become opportunistic invaders. In doing so, they threaten the delicate equilibrium of ecosystems by outcompeting native species and disrupting biodiversity (Rodríguez-Zaragoza, 1994; Samba-Louaka et al., 2019). This metamorphosis from harmless inhabitants to invasive aggressors highlights the dual nature of their adaptability, one that can be both awe-inspiring and alarming.

Venturing further into the realm of concern, the category of free-living amoebae (FLA) unveils some of the most notorious amoebic pathogens (Visvesvara and Stehr-Green, 1990; el-Fakahany et al., 1997; da Rocha-Azevedo et al., 2009; Thomas et al., 2010). These FLA, including species such as *N. fowleri*, *A castellanii*, and *B. mandrillaris*, carry the potential for devastation and death. *N. fowleri* colloquially dubbed the ‘brain-eating amoeba,’ strikes terror with its grim reputation– a mortality rate that reaches a staggering 97% (Güémez and García, 2021).

The emergence of *N. fowleri* as a human pathogen is nothing short of nightmares. Its entry into the human body typically occurs through the nasal passages while swimming in warm freshwater, and from there, it invades the brain, causing severe inflammation and destruction (Grace et al., 2015; Güémez and García, 2021). PAM, the condition caused by this relentless amoeba, progresses rapidly, often with a span of only a few days between initial symptoms and fatal outcomes (Ghanchi et al., 2017). The severity of the disease leaves little room for medical intervention to have a meaningful impact. *A. castellanii*, another member of the FLA, is proficient in causing chaos. Although its modus operandi differs from that of *N. fowleri*, the outcomes are similar. It can lead to a variety of infections in humans, affecting the eyes, skin, and central nervous system (Martinez, 1991). *Acanthamoeba* keratitis, an eye infection, is particularly troubling because it can result in vision loss or even blindness if left untreated (Imam and Mahgoub, 2008). *B. mandrillaris* reveals yet another alarming and deadly aspect of amoebic infections. This rare but devastating pathogen causes granulomatous amoebic encephalitis (GAE), a brain infection that often proves fatal. Its ability to evade the immune system and persist undetected for long periods makes it particularly dangerous, adding to the growing concern about amoebic diseases in human health (Matin et al., 2008; Siddiqui and Khan, 2015).

## 4 The beneficial aspect of amoebae

Amoebae play a crucial role in nutrient cycling, ecosystem balance, and have potential applications in medical and biotechnological research. The relationship between amoebae and bacteria can range from mutual to parasitic interactions (Shi et al., 2021). Phagocytosis is the process by which a living cell engulfs other cells or particles. Amoeba feed on bacteria, fungi, or other protists by engulfing them by phagocytosis into their digestive vacuoles and thus, amoebae act as predators of microorganisms (Van der Henst et al., 2016). Amoebae can form various relationships with other microorganisms (**Figure 1B**). There were three primary categories of interactions between amoebae and different bacteria, as summarized in **Table 1**. These interactions are: i) Predation: in this interaction, amoebae act as predators, preying on other microorganisms as their primary food source (Anscombe and Singh, 1948; Eisenberg et al., 1989; Van der Henst et al., 2016; Brock et al., 2018; Bornier et al., 2021; Shi et al., 2021); ii) Parasitism: amoebae engage in parasitism when they encounter pathogenic bacteria, which can replicate within the amoeba, potentially causing harm, and can also escape into the amoeba’s cytosol and the surrounding environment (Bozue and Johnson, 1996; Greub and Raoult, 2004; Thomas et al., 2006; Cervero-Aragó et al., 2014; Delafont et al., 2014; Shi et al., 2021; Price et al., 2024); iii) Mutualism: some amoebae have mutualistic relationships with other organisms (DiSalvo et al., 2015; Shu et al., 2018; Garcia et al., 2019). These endosymbionts provide benefits to amoebae while also benefiting from the association. These three types of interactions illustrate the dynamic relationships that amoebae can establish with various microorganisms in their environments.

**Table 1:** Summary of various type of interactions between Amoebae and bacteria.

Types of Interactions	Interaction between Amoebae and bacterial species		References
	Amoebae	Bacteria	
Predation	*Dictyostelium discoideum*	*Proteobacteria* (alpha, beta, gamma), *Bacteroidetes, Firmicutes, and Actinobacteria*	Anscombe and Singh, 1948; Eisenberg et al., 1989; Brock et al., 2018; Shi et al., 2021
Parasitic	*Acanthamoeba*, *Vermamoeba*, *Echinamoeba* *Protacanthamoeba*	*Legionella pneumophila, Mycobacterium llatzerense* *M. chelonae*, *M. ulcerans*, *M. leprae*, *Chlamydia trachomatis, Chlamydia pneumoniae*	Bozue and Johnson, 1996; Thomas et al., 2006; Delafont et al., 2014; Price et al., 2024
Mutualistic	*Dictyostelium discoideum*	*Burkholderia hayleyella Burkholderia agricolaris*	DiSalvo et al., 2015; Shu et al., 2018; Garcia et al., 2019

**Figure 2:**
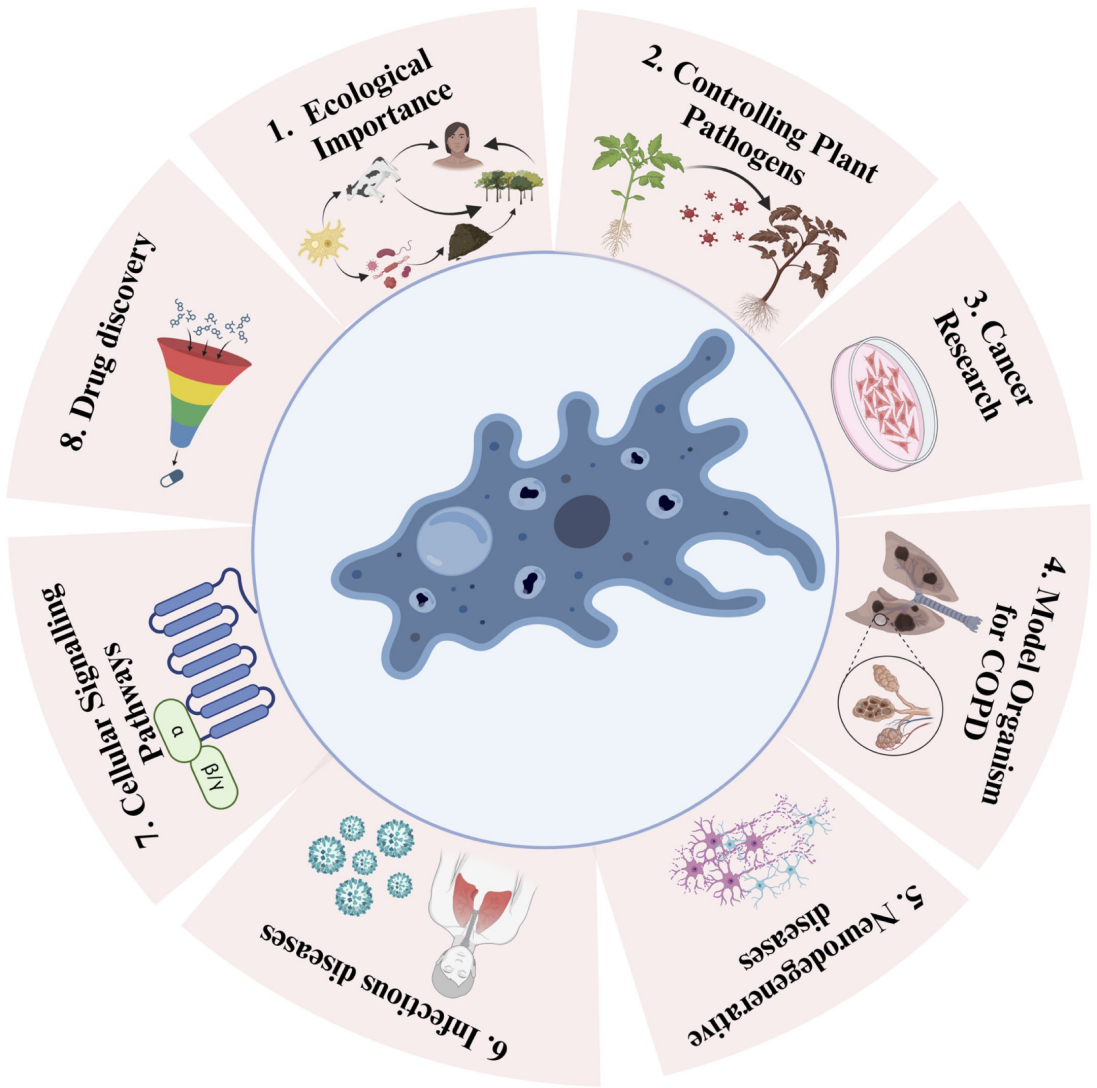
Schematic depiction of the versatile applications of amoebae and their positive impacts. Utilizing Amoebae for 1. Ecological Importance (e.g., *Acanthamoeba sp*, *Dictiostelium sp, Vermamoeba sp*), 2. Controlling Plant Pathogens (e.g., *Acanthamoeba sp*, *Dictiostelium sp*), 3. Cancer Research (e.g., *Dictiostelium discoideum*, *Acanthamoeba sp*), 4. Model in and Chronic Obstructive Pulmonary Disease (COPD) Studies (e.g., *Dictiostelium discoideum*), 5. Neurodegenerative diseases (e.g., *Dictiostelium discoideum*), 6. Infectious Diseases (e.g., *Dictiostelium discoideum*), 7. Cellular Signaling Pathways (e.g., *Dictiostelium sp*), and 8. Drug Discovery (e.g., *Amoeba proteus*).

There is evidence that amoeba act as a training ground for human pathogens that support the growth of human pathogens, such as *Legionella pneumophila* (Molmeret et al., 2005). On the other hand, amoebae show parasitic interactions with human pathogenic bacteria via phagocytosis (Rodríguez-Zaragoza, 1994). This interaction between amoebae and microorganisms is useful in biomedical research. These amoebae can resist bacteria that are pathogenic to humans (Greub and Raoult, 2004). Phagocytosis can be influenced by prey size, the presence of surface molecular patterns, hydrophobicity, seasonal variation, and prey density. To protect against phagocytosis, the pathogens produce extracellular capsules. This phenomenon was confirmed by *Klebsiella pneumoniae* forming a capsule against *D. discoideum* amoeba (Rodríguez-Zaragoza, 1994). Therefore, pathogens form the same capsule to prevent phagocytosis by mammalian cells. Clinical isolates of *K. pneumoniae* and *Acinetobactor baumannii* are known to show thick capsules and polysaccharide capsule formation. During the experiment, a mutated mucoid strain was introduced to assess amoeba (Bornier et al., 2021). Mutation in the auto-kinase domain of Wzc caused regulatory defects in capsule production, but this did not make the process of isolation of amoeba from the bacteria difficult. It was thought earlier that capsule formation protects pathogens from phagocytosis (Bornier et al., 2021). Subsequently, it was shown that the capsule provided partial protection. Capsules altered the predation kinetics of amoeba. Bactericidal activity differed among the different amoeba isolates. Bactericidal activity means how fast amoeba can kill a bacterial population. Isolates of *Vermamoeba vermiformis* required 24 h to kill the bacterial population, whereas isolates of *Acanthamoeba* required almost 48 h to kill the bacterial population (Fouque et al., 2015). Isolates of *Tetramitus* and *Vahlkampfia* could reduce the bacterial population by first–10-100 folds but after some time, their bactericidal activity stopped, causing equilibrium. Free-living amoebae thrive in the natural environment, mainly in the soil; thus, they can be exposed to bacterial complexes and can discriminate and feed on specific bacteria. Our natural environment acts as a rich source of diverse amoeba with broad-spectrum predatory activities against human pathogens (Bornier et al., 2021). Amoebae have been increasingly recognized as a valuable model for studying various human diseases (Thewes et al., 2019). Despite the vast evolutionary distance between amoebae and humans, they share many cellular processes and genetic pathways, making them relevant models for investigating disease mechanisms and potential therapeutic targets (Eichinger et al., 2005). This article discusses the use of amoebae as a model for different human diseases and highlights the insights gained from these studies (**Figure 2**).

### 4.1 Ecological importance

Amoebae play an essential role in nutrient cycling and ecological balance by participating in decomposition, regulating microbial populations, supporting soil health, and maintaining food webs in both terrestrial and aquatic environments (Clarholm, 1981). Amoebae are vital decomposers that consume bacteria, fungi and organic material, breaking down complex organic matter into simpler nutrients, which are then released into the soil and aquatic systems. This nutrient release is critical for supporting primary producers like plants and algae (Clarholm, 1981). These processes contribute to nutrient cycling by facilitating the recycling of nitrogen, phosphorus, and other essential nutrients, which are necessary for ecosystem productivity (Wilkinson, 2008). Amoebae also act as predators of bacteria and other microorganisms, helping to regulate microbial populations. By preventing any single microbial species from becoming overly dominant, amoebae help maintain microbial diversity, which is essential for ecological resilience and nutrient dynamics (Rodríguez-Zaragoza, 1994). This predation helps control pathogenic bacteria populations as well, indirectly protecting plant and animal health (Bonkowski and Brandt, 2002). In soils, amoebae contribute to soil structure and fertility by consuming microbes and releasing nutrients into the rhizosphere (root zone), which benefits plant growth. Amoebae are also involved in symbiotic relationships with bacteria and fungi that enhance nutrient availability in the soil, playing a fundamental role in humus formation, which is essential for soil health (Rønn et al., 2002). This activity improves soil moisture retention and nutrient availability, which supports a balanced microbial (Adl and Gupta, 2006). In aquatic systems, amoebae are integral to the microbial loop, where they recycle nutrients by consuming bacteria and returning nutrients to the water. This nutrient recycling supports primary producers like phytoplankton, which form the base of aquatic food webs, linking microbial communities to larger organisms and ensuring the flow of energy throughout the ecosystem (Sherr and Sherr, 1988; Hwang and Heath, 1997). Amoebae populations can be indicators of environmental health due to their sensitivity to changes in environmental conditions, such as nutrient levels, pollution, and habitat disturbances. Changes in amoebae populations may signal shifts in ecosystem health, making them valuable bioindicators in both terrestrial and aquatic environments (da Silva et al., 2022). Analyzing the ecological balance of amoebae and their persistence can be studied by using Geographic Information Systems (GIS) and remote sensing technologies to assess the ecological balance of amoebae populations and their persistence in various environments (Carella et al., 2022; Viani et al., 2023). By leveraging spatial data and environmental indicators, this approach aims to identify suitable habitats, monitor distribution patterns, and pinpoint key environmental proxies that influence amoebae ecology. The integration of satellite imagery, digital elevation models, and spatial analysis can help delineate areas of high suitability, shedding light on how factors such as temperature, humidity, soil composition, and vegetation cover impact the survival and distribution of amoebae species (Orusa et al., 2020; Carella et al., 2022). This information is critical for understanding the environmental dynamics that support the presence of these organisms, with potential applications in ecosystem management, disease control, and conservation strategies.

### 4.2 Use of amoeba controlling plant pathogen

Plant pathogens are usually bacteria, viruses, fungi, oomycetes, or nematodes that can cause diseases in plants, leading to significant economic losses in agriculture and horticulture (Wang et al., 2022). Controlling plant pathogens requires various strategies depending on the specific pathogen and the affected crop (Pandit et al., 2022). Implementing good agricultural practices, such as crop rotation, proper irrigation, and balanced fertilization, helps maintain plant health and reduce susceptibility to pathogens (McDonald and Stukenbrock, 2016; Samaddar et al., 2021). Natural host-plant resistant plant varieties are an effective control strategy for managing these pathogens. Biological control, by introducing natural enemies or beneficial microorganisms, suppresses pathogen growth (Pandit et al., 2022). Although chemical control with fungicides and bactericides can be used, careful application is essential to avoid harming the environment and non-target organisms. The enforcement of quarantine measures prevents pathogens from spreading into new areas. The use of amoebae as a means of controlling plant pathogens has gained attention as a promising biological control strategy (Pandit et al., 2022). Certain amoebae, particularly those belonging to the group known as free-living amoebae (FLA), exhibit predatory behavior towards plant pathogens (Bornier et al., 2021). When introduced into soil or plant environments, these amoebae actively feed on pathogenic microorganisms, thereby suppressing their growth. By preying on harmful fungi, bacteria, and other plant pathogens, amoebae help to maintain plant health and reduce the risk of diseases (Long et al., 2018, 2019). This eco-friendly approach offers a sustainable alternative to chemical control methods, minimizing environmental impacts, and safeguarding non-target organisms. Although research in this area is ongoing, harnessing the potential of amoebae as biocontrol agents holds promise for enhancing agricultural practices and crop protection (Rodríguez-Zaragoza, 1994; Long et al., 2019). The soil-borne fungus *Rzioctonia solani* can affect many important crop plants, including rice, where it causes sheath blight disease. It is a necrotic disease of rice that can be identified by lesions initiating at the apical ends of rice sheaths. A significant interaction between FLA and *Rhizoctonia solani* was observed by scanning electron microscopy, which helped to suppress the growth of fungal mycelia (Long et al., 2019). A bacteria called *Xanthomonas oryzae* causes bacterial blights in rice (Jain et al., 2023). According to previous studies, amoeba-bacterial interactions can lead to the biocontrol of plant pathogenic bacteria in plants. Five different amoebae were used for this study: *Acanthamoeba polyphaga, A. lenticulata, A. castellanii, Vermamoeba vermiformis* and *D. discoideum* with two pathovars of *X. oryzae.* The study reported that *A. lenticulata, A. polyphaga, V. vermiformis* and *D. discoideum* significantly reduced the number of bacteria after 24 h, and *A. lenticulata* and *V. vermiformis* had the strongest effect on pathogenicity. These amoebae lyse bacterial cells not through phagocytosis but by secreting some toxic compounds. However, sometimes *X. oryzae* forms a bacterial biofilm as a defense mechanism that cannot be degraded by the amoebae (Long et al., 2018).

### 4.3 Amoeba as a model in cancer research

Amoebae, particularly *D. discoideum*, have been employed in cancer research because of their ability to undergo a unique process called chemotaxis, which resembles the movement of cancer cells toward chemotactic gradients (Roussos et al., 2011). This similarity allows researchers to study cancer cell migration and invasion. Additionally, amoeba models have been employed to investigate the effects of potential anticancer drugs on cell migration and invasion, aiding in the identification of novel therapeutic agents (Roussos et al., 2011). To understand the process of differentiation or de-differentiation in the context of genes, proteins, and the environment for therapeutic and diagnostic uses, evolutionary lower organisms, such as *Acanthamoeba* can be used because they show cellular differentiation and de-differentiation properties that can explain the transition of normal cells to dormant cells in the primary stage of research (**Figure 1B**). The FLA contains two stages in its life cycle, a trophozoite stage where they are metabolically active and reproduce by mitotic division, and a cyst stage where they have minimal metabolic activity. Cyst formation occurs when the environment is unfavorable, and the return of favorable environmental conditions encourages cyst de-differentiation into the trophozoite form (Ahmed Khan et al., 2015). *D. discoideum* is an amoeba that has been used to study cancer-associated genes. Phosphatase and tensin homolog (PTEN) in cancer cells is one of the most frequently mutated tumor suppressor genes (Viennet et al., 2023). PTEN plays a crucial role in both cancer and amoeba cells (Chow and Baker, 2006). In cancer cells, mutations or alterations in PTEN can lead to uncontrolled cell growth and division, contributing to the development and progression of cancer. In amoeba cells, PTEN is involved in regulating cell growth and survival, similar to its role in normal mammalian cells. It helps maintain proper cell function and prevents excessive cell proliferation (Nguyen et al., 2014). The study of PTEN in both cancer and amoeba cells is important for understanding cellular processes, disease mechanisms, and potential therapeutic targets in cancer treatment (Nguyen et al., 2014; Mathavarajah et al., 2021). There are similarities between *Entamoeba* and cancer cells in the formation of Multinucleated Giant Cells (MGCs) and polyploid cells, making amoebae a promising model system for studying polyploidy in the context of cancer (Manna et al., 2020a, 2020b; Hazra and Manna, 2023; Hazra et al., 2023).

### 4.4 Model organism for chronic obstructive pulmonary disease

COPD is a complex respiratory disease that primarily affects humans and is often caused by smoking and exposure to pollutants. Scientists typically use animal models or *in vitro* cell cultures to study COPD and to develop potential therapies (**Figure 1B**). Recently, they discovered that the social amoeba *D. discoideum* reacts with cigarette smoke or cigarette smoke extract (CSE). Therefore, they can be used as models to identify new potential ways to adopt preventive strategies that will be helpful in the protection of airways against cigarette smoke (Wu et al., 2020; Ghosh et al., 2022). In COPD, as airway remodeling is linked to mitochondrial dysfunction, overexpression of canonical inner mitochondrial membrane transport proteins measured in the cDNA library can increase mitochondrial metabolism and protect against CSE (Kliment et al., 2021).

### 4.5 Amoeba in neurodegenerative disease research

While amoebae are considerably simpler than humans and lack a nervous system, they offer a unique platform for understanding fundamental cellular processes, particularly those related to protein aggregation, cellular stress responses, and mitochondrial dysfunction, which are central to the pathogenesis of neurodegenerative diseases. Several amoebae species share molecular and functional similarities with the neurons in the human brain. Researchers have utilized these amoebae models to study neurodegenerative diseases such as Alzheimer’s and Parkinson’s diseases (Haver and Scaglione, 2021; Storey et al., 2022). One of the key hallmarks of neurodegenerative diseases, such as Alzheimer’s, Parkinson’s, and Huntington’s, is the misfolding and aggregation of specific proteins (e.g., amyloid beta, alpha-synuclein, and huntingtin). Amoebae can be engineered to express proteins related to human diseases. This enables researchers to study the mechanisms underlying protein misfolding and aggregation in a simplified and controlled cellular environment. By expressing human disease-related proteins in amoebae, scientists can examine the pathological effects of these proteins on cellular function and viability, thereby providing valuable insights into disease mechanisms and potential treatment strategies (Santarriaga et al., 2015). Amoebae provide insight into how cells respond to stress. Neuronal cells in neurodegenerative diseases are often exposed to various stressors. Understanding how amoebae cope with stress can illuminate the pathways and mechanisms involved in cellular stress responses, which may be relevant to neurodegenerative conditions.

### 4.6 Amoeba as a host for infectious diseases studies

Amoebae, single-celled microorganisms often found in soil and water, have emerged as valuable models for the study of infectious diseases. Although they are relatively simple organisms, amoebae exhibit a surprising degree of sophistication in their interactions with pathogens, making them an excellent model system for understanding the fundamental principles of infectious disease dynamics, pathogen evolution, and host-pathogen interactions. Amoebae can serve as hosts for various pathogens, including bacteria and parasites, allowing researchers to study infectious diseases such as Legionnaires’ disease and amoebic dysentery (Shi et al., 2021). Amoebae can be infected by a wide range of pathogens including bacteria, viruses, fungi, and protozoa. Studying these interactions within amoebae provides insights into the initial stages of infection, mechanisms by which pathogens invade host cells, and how amoebae respond to these threats. Many pathogens that infect amoebae replicate within the host cell, similar to the way they infect higher organisms. This allows researchers to investigate the strategies employed by pathogens to evade the host immune system and manipulate host processes to ensure their survival and reproduction. This information can be extrapolated to understand more complex host-pathogen interactions in higher organisms. The interactions between amoebae and these pathogens shed light on the mechanisms of infection, pathogen survival, and immune evasion (Uribe-Querol and Rosales, 2020). Moreover, Amoebae can be used to study the evolution of virulence in various pathogens. As amoebae represent a simplified host environment, researchers can explore how pathogens evolve to become virulent in response to selective pressure. This can shed light on the broader patterns of virulence evolution in infectious diseases. In addition, the study of amoeba-resistant bacteria offers potential targets for developing new antibiotics (Oh et al., 2001).

### 4.7 Amoeba in cellular signaling pathway research

Despite their simplicity as single-celled organisms, amoebae possess conserved cellular signaling pathways that share remarkable similarities with those found in human cells. This similarity has made amoebae a valuable model for studying basic cellular functions and the roles of these pathways in human diseases (Shi et al., 2021). One example of the significance of studying these pathways in amoebae is the exploration of G protein-coupled receptors (GPCRs). GPCRs are a diverse family of cell surface receptors that play pivotal roles in a wide array of human diseases, including cardiovascular disorders and cancer. In amoebae, GPCRs are involved in crucial cellular processes, and their study has provided insights into the fundamental mechanisms underlying receptor signaling (Brewer et al., 2013; Pergolizzi et al., 2017). By understanding how these conserved pathways function in amoebae, researchers can gain a deeper understanding of their roles in human cells and their involvement in disease onset and progression. This knowledge offers a unique perspective on the potential therapeutic targets and strategies for a range of medical conditions. Consequently, the study of these conserved signaling pathways in amoebae has significant implications for advancing our understanding of human health and the development of treatments for diseases involving GPCR, such as cardiovascular disorders and cancer (Brewer et al., 2013; Pergolizzi et al., 2017).

### 4.8 Amoeba in drug discovery

Amoebae are incredibly adaptable and have been extensively studied in biology and genetics. One of the key advantages of using amoebae in drug screening is their ability to replicate certain aspects of cellular functions, making them an excellent surrogate for more complex organisms, such as humans. The amoeba model provides a cost-effective and efficient platform for drug screening. Researchers can test thousands of compounds for potential therapeutic effects against various diseases using amoeba-based assays (Duez et al., 1991). Amoeba-based assays involve exposing microorganisms to a wide range of chemical compounds to assess their potential therapeutic effects. These assays can be tailored to study specific diseases, and the screening process is highly versatile. Researchers can modify and optimize the assay conditions to mimic the cellular environment relevant to the disease being studied. This adaptability is a key factor that makes amoebae models invaluable for drug discovery. Thousands of chemical compounds were tested using a typical amoeba-based assay (Kangussu-Marcolino et al., 2019). These compounds include existing drugs, natural products, or new molecules that are synthesized specifically for drug discovery (Manna et al., 2010, 2013). Amoebae were exposed to these compounds, and their responses were carefully monitored and analyzed. This process allows researchers to identify compounds that have potential therapeutic effects against target diseases. Such assays can be high-throughput, enabling simultaneous screening of a large number of compounds (Harrison et al., 2015) (**Figure 2**). With the growing interest in natural compounds, the potential for discovering novel drugs or treatments from amoebae is high. Exploring their secondary metabolites and understanding their interaction with other microorganisms could lead to breakthroughs in the development of antimicrobial and anti-cancer therapies. Amoebae exhibit intriguing similarities with macrophages, which are immune cells responsible for the critical role of engulfing and destroying pathogens within the body (Esteban et al., 2015). Therefore, amoebae can be used to study host-pathogen interactions and immune responses (Thewes et al., 2019). The parallelism between the roles of amoebae and macrophages as phagocytes is remarkable. Both cell types are capable of engulfing and eliminating pathogens. The similarities in their cellular processes provide researchers with an opportunity to investigate host-pathogen interactions in a simplified yet informative model. Through amoeba-based studies, scientists have gained insights into how pathogens are recognized, internalized, and processed within host cells. These investigations offer a valuable perspective on the immune response, shedding light on how immune cells, including macrophages, combat infection. The findings from amoeba models can be translated into a better understanding of the molecular and cellular mechanisms underlying human immune responses, thereby contributing to the broader field of immunology. This knowledge contributes to a better understanding of infectious diseases and the development of new strategies to combat infections (Shi et al., 2021). In conclusion, the use of amoebae as models for different human diseases has proven to be a powerful tool in biomedical research. Their genetic and functional similarities with human cells allow scientists to investigate disease mechanisms, perform drug screenings, and gain valuable insights into host-pathogen interactions (Thewes et al., 2019; Samba-Louaka, 2021). As our understanding of amoebae biology and genetics continues to advance, their potential as model organisms for studying human diseases is likely to expand, contributing significantly to advancements in medical research and healthcare (Haver and Scaglione, 2021) (**Figure 2**).

## 5 Concluding and future perspective

Amoebae are a double-edged sword, offering significant ecological and biomedical benefits while posing serious health risks. Amoebae play a fascinating and multifaceted role in biology, ecology, and human health. This review sheds light on their diverse characteristics, encompassing both positive ecological contributions and potential threats to ecosystems and human well-being (Shi et al., 2021). In practical applications, amoebae have shown promise as a means of controlling plant pathogens in agriculture. Their predatory behavior towards plant pathogens offers an eco-friendly and sustainable alternative to chemical control methods, safeguarding the environment and non-target organisms (Viani et al., 2023). Furthermore, amoebae have emerged as valuable models for studying various human diseases, including cancer, neurodegenerative diseases, infectious diseases, and cellular signaling pathways. Their genetic and functional similarities to human cells make them relevant tools for investigating disease mechanisms and potential therapeutic targets (Haver and Scaglione, 2021). In wastewater management, amoebae demonstrate their potential as efficient biodegraders of pollutants, effective removal of pathogens, and contributors to sludge reduction. Their inclusion in wastewater treatment systems offers cost-effective and eco-friendly solutions, promotes sustainable methods for reducing environmental pollution, and reuses treated water. Balancing these positive and negative aspects are crucial for coexisting amoebae, and ongoing research promises advancements in utilizing their unique properties for ecological restoration, human health, and wastewater management. As we deepen our understanding of these organisms, the challenge will be to minimize their harmful impacts while maximizing their beneficial roles. Future research in amoebae biology, together with advancements in technology, offers promising potential for developing new therapeutic strategies, improving ecosystem management, and enhancing human health. Continued interdisciplinary efforts will be essential in harnessing the full potential of Amoebae in a way that benefits society as a whole.
